# EXACT2: the semantics of biomedical protocols

**DOI:** 10.1186/1471-2105-15-S14-S5

**Published:** 2014-11-27

**Authors:** Larisa N Soldatova, Daniel Nadis, Ross D King, Piyali S Basu, Emma Haddi, Véronique Baumlé, Nigel J Saunders, Wolfgang Marwan, Brian B Rudkin

**Affiliations:** 1 Department of Computer science, Brunel University, Kingston Lane, Middlesex, London, UB83PH, UK; 2 Centre for Systems and Synthetic Biology, Brunel University, Kingston Lane, Uxbridge, London, UB83PH, UK; 3 Directed Connections Ltd., 59 Elm Street, New Haven, CT, 06510, USA; 4 Manchester Institute of Biotechnology, the University of Manchester, 131 Princess Street, Manchester, M13 9PL, UK; 5 Magdeburg Centre for Systems Biology, Otto von Guericke University MagdeburgPfälzerstr. 5, Magdeburg, 39106, Germany; 6 Laboratory of Molecular Biology of the Cell, CNRS, Ecole Normale Supérieure de Lyon, University of Lyon 1, 46 allée d¹Italie, Lyon, 69364, France

## Abstract

**Background:**

The reliability and reproducibility of experimental procedures is a cornerstone of scientific practice. There is a pressing technological need for the better representation of biomedical protocols to enable other agents (human or machine) to better reproduce results. A framework that ensures that all information required for the replication of experimental protocols is essential to achieve reproducibility.

**Methods:**

We have developed the ontology EXACT2 (EXperimental ACTions) that is designed to capture the full semantics of biomedical protocols required for their reproducibility.

To construct EXACT2 we manually inspected hundreds of published and commercial biomedical protocols from several areas of biomedicine. After establishing a clear pattern for extracting the required information we utilized text-mining tools to translate the protocols into a machine amenable format. We have verified the utility of EXACT2 through the successful processing of previously 'unseen' (not used for the construction of EXACT2) protocols.

**Results:**

The paper reports on a fundamentally new version EXACT2 that supports the semantically-defined representation of biomedical protocols. The ability of EXACT2 to capture the semantics of biomedical procedures was verified through a text mining use case. In this EXACT2 is used as a reference model for text mining tools to identify terms pertinent to experimental actions, and their properties, in biomedical protocols expressed in natural language. An EXACT2-based framework for the translation of biomedical protocols to a machine amenable format is proposed.

**Conclusions:**

The EXACT2 ontology is sufficient to record, in a machine processable form, the essential information about biomedical protocols. EXACT2 defines explicit semantics of experimental actions, and can be used by various computer applications. It can serve as a reference model for for the translation of biomedical protocols in natural language into a semantically-defined format.

## Background

The standardization of experimental protocols is at the heart of GLP (good laboratory practice) and GMP (good manufacturing practice) procedures [1]. These have been essential for generating data required by regulatory agencies for many years. A high-degree of rigor is essential to ensure the reproducibility and relevance of the observations on which experimental conclusions are based.

With the increasing complexity of experimental methods there is an increasing technological need for the representation of biomedical protocols in a way that ensures that sufficient and unambiguous information is recorded to enable another agent (human or machine) to replicate these protocols. The EXACT2 (EXperimental ACTions) ontology reported in this manuscript provides a representation of experimental protocols that ensures their reproducibility and is easily processable by computer programs.

### Related projects

Several projects have contributed to the development of explicit semantically defined representation of biomedical protocols. The Ontology for Biomedical Investigations (OBI) project is developing an integrated ontology for the description of biological and clinical investigations [2]. This ontology aims to support the consistent annotation of biomedical investigations, and represents the design of investigations, the protocols and instrumentation used, the material used, the data generated, and the type of analysis performed. OBI is a valuable resource for recording information about biological and clinical assays, their designs, inputs and outputs. OBI also defines such terms relevant to the description of protocols as *protocol, investigator, documenting*, and *data transformation*.

However, OBI has not been designed to capture all essential information about experimental procedures; EXACT2 aims to address this need. The OBI representation is complicated, and design decisions may lead to a combinatorial explosion in the size of the representation. For example, the class *OBI: storage *has such subclasses as *OBI: agar stab storage, OBI: anticoagulant tube storage of blood specimen, OBI: paraffin storage*. One can see that a vast number of classes would be required to represent the storage of every possible type of biochemical entity and a piece of equipment used in labs. Instead, EXACT2 defines only one class *EXACT2: store *with a limited number of the required descriptors, including *biochemical entity *and *equipment *instances of which are imported from external resources. The EXACT2 design philosophy is to aim to provide as simple as possible representations of biomedical protocols.

Taverna is a domain-independent workflow management system - a suite of tools used to design and execute scientific workflows and aid *in silico *experimentation [3]. Taverna enables the linking together of scientific resources, error handling, service invocation, data streaming, and provenance tracking. Taverna is a popular system and is used by many projects including Ondex for data integration and visualisation [4], e-LICO for interdisciplinary collaborative research in data mining and data-intensive science [5] and next generation sequencing [3].

Taverna considers a biomedical protocol to be a workflow, but it does not provide a rigorous logically defined representation of protocols. Instead it provides a high-level declarative way of specifying what a particular *in silico *experiment modelled by a workflow is designed to achieve, not *how *it will be executed; see for example a protocol at http://www.myexperiment.org/workflows/387. EXACT2 representations are complementary to Taverna and focus on *how *experiments should be executed.

In conclusion the representations of biomedical protocols provided by EXACT2 are orthogonal and complimentary to other relevant representations, e.g. workflows in Taverna and investigations in OBI.

## Methods

We judged that manual analysis of actions would yield higher quality results compared with text mining methods. Therefore we manually inspected hundreds of published and commercial biomedical protocols from multiple areas of biomedicine, including neurology, epigenetics, metabolomics, stem cell biology, etc. [6]. We analysed instructions, notes, alerts, properties of experimental actions, what conditions are required and what goals are specified. We noticed after several rounds of analyses that newly considered protocols, even from different areas, did not add much information to that already formalised in EXACT2 knowledge. We therefore concluded that the representation of experimental actions in the scientific literature is limited and relatively consistent. There are of course differences in lexical patterns used to express information about experimental actions, but the number of distinct experimental actions mentioned in protocols is surprisingly low (<100). The language used to describe protocols is also considerably restricted compared with natural language used in other texts. As such, this apparent 'simplicity' would be expected to offer a consistent and reproducible means for presenting protocols.

We then updated the previous version of EXACT [7] using the entities and relation identified to be relevant to the representation of experimental procedures. We did this using the ontology editor Protégé 4.3, and the reasoner HermiT 1.3.8. to detect inconsistencies. There are a number of substantial changes in EXACT2 compared to EXACT (see the summary of changes in the results section), but the most important is the addition of descriptors to each experimental action. The reason for this is that it is not possible for biologists to safely reproduce a biomedical procedure without knowing the values of such descriptors as *temperature, equipment, duration *of experimental actions. Unfortunately, such information is frequently missing in published protocols, or is inconsistent between different protocols, leaving interpretation up to the reader and therefore dependent on personal or collective experience. For example, vital information is missing from this description of experimental actions:

"Reconstitute bFGF and EGF with 0.1% BSA solution at a concentration of 100 μg/mL. You will need 20 μL of each per 100 mL of complete medium. Freeze unused portions in aliquots".

The liquid component of the 0.1% BSA (Bovine Serum Albumin) solution is not identified (it can be distilled water, millipore water, phosphate-buffered saline, etc.). Furthermore, these actions must be performed under sterile conditions, otherwise experiments using these materials may fail. To overcome this difficulty we consulted with experts in various biomedical areas to identify what descriptors are required, and what descriptors are optional, for each experimental action included in EXACT2.

Following OBO Foundry recommendations (see [8]), EXACT2 imports classes and relations from external resources. We employed the principle of MIREOT (Minimum Information to Reference External Ontology Terms) for consistent reference of external terms [9]. MIREOT requires the inclusion of the following information: (1) source ontology URI (Unique Resource Identifier); (2) source term URI; and (3) target direct superclass URI. We used the OntoFox web application to import external terms to EXACT2 [10]. In total, 25 terms were imported into EXACT2 from BFO, OBI, IAO, PATO (see the section below for more detail).

EXACT2 is encoded in OWL-DL, a language widely used by the research community and recommended by OBO Foundry. We used github for versioning and depositing EXACT2. The latest version of the ontology and files with external imports are available at: https://github.com/larisa-soldatova/EXACT. EXACT2 is also available at BioPortal: http://bioportal.bioontology.org/ontologies/EXACT.

## Results

### EXACT2

We present a fundamentally new version of the ontology EXACT2 for recording biomedical protocols. EXACT2 aims to explicitly define the semantics of experimental protocols in order to ensure their reproducibility, and to support computer applications that assist biologists in the preparation, maintenance, submission and sharing of experimental protocols. The range of experimental procedures in biomedicine is extremely wide, and ever increasing. While EXACT2 aims to cover the majority of experimental actions found in biomedical protocols, our estimate is that EXACT2 currently includes 85% of typical experimental actions. This estimate is based on processing of previously 'unseen' protocols. The scope of EXACT2 is restricted, for example by not allowing negations. Negations are rarely used in biomedical procedures, and are problematic to represent under the open world assumption. Our aim is to keep EXACT2 as simple as possible, and consequently such instructions as *do not smoke *cannot be represented with EXACT2, but such information can be captured in a form of notes as free text.

It is a challenging task to capture and formalise information pertinent to biomedical protocols, we therefore applied a modular approach to the problem. EXACT2 is focused on the formal description of experimental actions and imports other entities participating in experimental actions from external resources such as ChEBI (Chemical Entities of Biological Interest) dictionary [11] for biochemical entities and eagle-i (see the eagle-i project [12]) ontology for experimental equipment.

### Upper level ontologies

**Figure 1:**
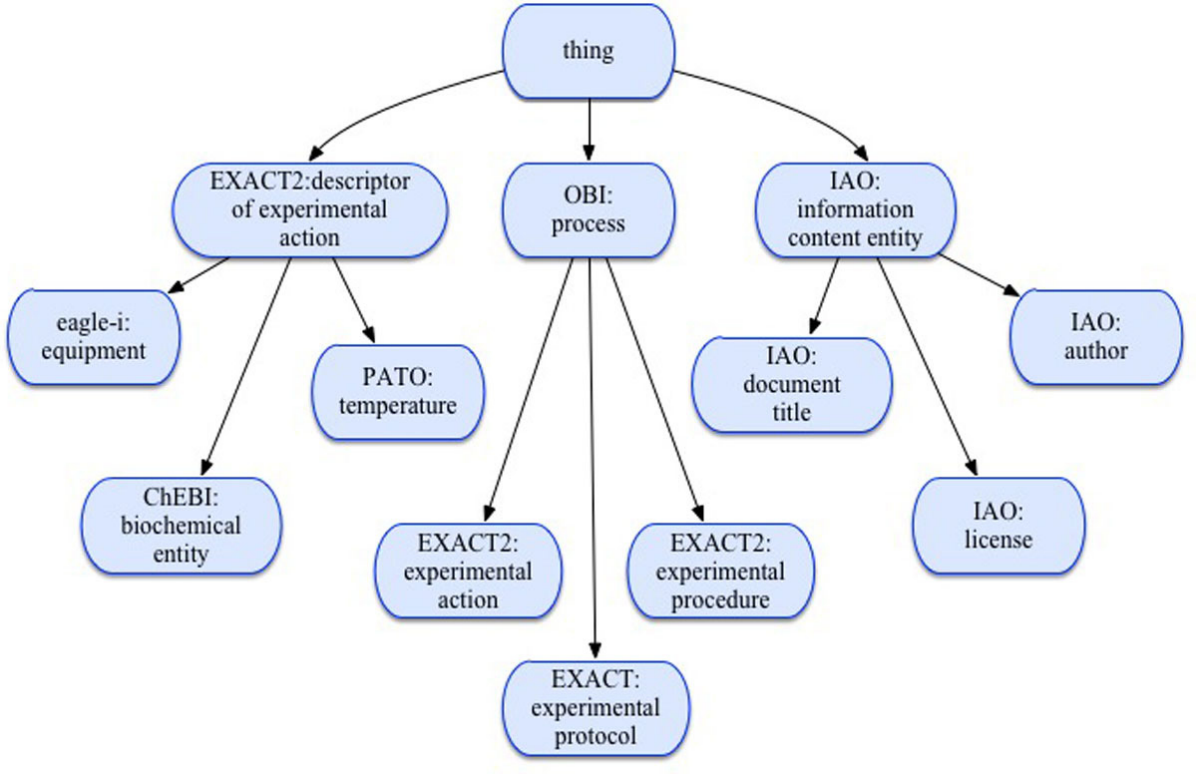
**The EXACT2 upper level classes (a fragment)**. EXACT2 has the following upper level classes: *process *(i.e. experimental actions, procedures and protocols), *descriptor of experimental action *(i.e. equipment, biochemical entities, temperature, speed, volume, etc.) and *information content entity *(i.e. author, licence, etc.).

The previous version of EXACT used SUMO (the Suggested Upper Merged Ontology) [13] and Time Ontology (see [14]) as upper-level ontologies [7]. Following OBO Foundry recommendations, the new version has been constructed with the use of the top-level classes from BFO (the Basic Formal Ontology) 1.1 [15], IAO (the Information Artifact Ontology) [16], PATO (Phenotype And Trait Ontology) [17] and OBI [2] (see Figure 1). The result is that the class *SUMO: Object *has been replaced with the class *OBI: material entity*, and the class *EXACT: proposition *has been replaced with the class *IAO: information content entity*. EXACT2 imports the PATO classes: *volume, speed, temperature *as descriptors of experimental actions. Such classes as *IAO: document title, IAO: author identification *were imported to EXACT2 to enable the representation of protocol's provenance. IAO classes *textual entity *and *table *were imported to capture information about such important textual elements of biomedical protocols as tables, notes, cautions, troubleshooting.

The adherence to BFO, IAO, PATO and OBI enables an efficient integration of EXACT2 with other biomedical ontologies, particularly with ChEBI for the representation of biochemical entities, and eagle-i for the representation of equipment used in experiments.

### Structure of EXACT2

EXACT2 has a streamlined structure in order to ease the navigation through the ontology hierarchy. Underused top level classes such as *EXACT: mode of transformation, EXACT: mode of separation *have been deprecated. We have also deprecated classes that had only one or two subclasses. For example the class *EXACT: shake *had only one subclass *EXACT: swirl*, the class *EXACT: cover *had only one subclass *EXACT: seal *and the class *EXACT: remove *had only two subclasses *EXACT: vortex *and *filter*. All these classes are now defined as subclasses of the class *EXACT2: experimental action*. Consequently, a user or a computer application, in order to process information does not need to identify that, for example, the action *rotate *is a 'subtype of the *remove *type' of an action.

The structure of EXACT2 has been simplified further by the deprecation of roles. For example the class *EXACT: container *was represented as a role played by equipment. While this is an accurate representation, and different pieces of equipment can play different roles, we judged that EXACT2 should only include the top-level class *EXACT2: equipment *without specifying what roles it can play or what functionality it may have. Instead, a modular approach that enables an import of lab-specific equipment is employed for the encoding of biomedical protocols.

EXACT2 no longer directly supports commands (i.e. *stop, continue*) and other expressions (i.e. *if-then expression*) that could be included to biomedical protocols in order to describe a sequence of experimental actions. There are other formalisms (e.g. Petri nets) that are better suited for the representation of such knowledge.

### The experimental actions branch

The experimental actions branch has been significantly extended. The previously published version of EXACT contained 45 actions, including command actions, equipment setup actions, and data actions. 33 of the 45 actions were classified as experimental action. We manually analysed hundreds of biomedical protocols and added 51 experimental actions that were missing from the previous version. For example the actions *EXACT2: aliquot *(definition: an experimental action "to measure out (a substance) into small samples of equal size; to divide into aliquot parts, especially for use as experimental samples" [18]), *EXACT2: dilute *(definition: an experimental action "to make or become less concentrated, especially by adding water or a thinner, (of a solution, suspension, mixture, etc.) having a low concentration or a concentration that has been reduced by admixture" ([19])were added to the new version.

EXACT2 imports three actions from OBI. Specialists in the area of biomedicine analysed the OBI branch *planned process *and identified OBI classes that represent experimental actions. This resulted in the addition of such classes to EXACT2 as *OBI: elution *(definition: the process of extracting one material from another by washing with a solvent to remove adsorbed material from an adsorbent (as in washing of loaded ion-exchange resins to remove captured ions)), *OBI: injection *(definition: injection is process which aims at introducing a compound or a mixture into a material entity (either biological entity or instrument) by relying on devices such as syringe or injector connection, attached or forced into a vascular system (veins of an organism or tubes of a machine) or in a tissue.).

### Mapping of EXACT2 experimental actions to OBI planned processes

EXACT was developed before OBI independently included semantic descriptors relevant to experimental actions. As a result there are several EXACT and OBI classes that have similar semantic meanings. Following ontology design best practices EXACT2 explicitly maps such classes *via *the annotation property *has synonym*. For example the class *EXACT2: wait *is mapped to *OBI: waiting*, the class *EXACT2: store *is mapped to *OBI: storage*. The semantic meaning of these processes is similar, but not identical. In EXACT2 these actions are defined *via *a set of descriptors. The experimental action *EXACT2: store *requires the recording of such descriptors as (storage) temperature, period (of storage), biochemical entity (to be stored), (storage) condition, and equipment (used for storage). Otherwise, according to EXACT2, this experimental action cannot be reproduced adequately. OBI has the following properties for the process *storage*: *has specified input some material entity *(this is consistent with the EXACT2 descriptors *biochemical entity *and *equipment*), *achieves planned objective some 'material maintenance objective' *(based on our analysis of the protocols, EXACT2 does not enforce the recording of the descriptor *goal *for this experimental action), and *realizes some (concretizes some 'plan specification') *(again, based on our analysis of the protocols, EXACT2 does not enforce the description of the plan specification). Thus, OBI lacks the representation of such essential properties of the process *storage *as (storage) temperature, and period (of storage). Some biochemical entities must be stored at (or below) -196°C, -80°C, -20°C, +4°C and some may be kept at a room temperature. The failure to record such essential information may result in the failure to correctly follow biomedical procedures, and produce erroneous results. It is true that a storage period is frequently not specified in biomedical protocols. However, it is important information, for example for safety and reproducibility, and it is essential to record it whenever possible.

### Optional descriptors

One of the requirements for EXACT2 is to represent which descriptors of experimental actions are essential, and which are optional. In a scenario where a user submits a protocol to an EXACT2-based system, and some experimental actions in that protocol do not include essential descriptors, then the system will request that the user specifies those missing descriptors (see the next section for more explanations). Conversely, a frequent occurrence in protocols is that experimental actions contain descriptors that are non-essential (= optional). These descriptors are beneficial to the understanding of protocols, and therefore should be preserved in machine amenable representations of protocols. However, a system supporting such representations needs to be able to strike the right balance between ensuring that all essential information about a protocol is captured, and remaining user-friendly by not enforcing users to input non-essential information. For example, it is not essential to specify the value of the descriptor *temperature *for the actions *EXACT2: filter *and *EXACT2: resuspend*. These actions are typically executed at *room temperature*, or at the temperature of the previous step, and it is normally specified in protocols if otherwise. EXACT2 aims to represent typical situations and, in order not to enforce the recording of the descriptor *temperature *for every instance of the classes *EXACT2: filter *and *EXACT2: resuspend*, EXACT2 needs to classify this descriptor as optional.

Unfortunately, the limited expressivity of OWL does not allow us to represent that an experimental action **may **have certain descriptors. To overcome this limitation we have introduced the class *EXACT2: optional descriptor of experimental action *with such subclasses as *EXACT2: (optional) temperature, EXACT2: (optional) equipment*, etc. An alternative solution would have been to assign probabilities to the statements 'an experimental action has a descriptor' [20]. However, we judged that the probabilistic approach would unnecessarily complicate the EXACT2 representations.

## Use case: translation of biomedical protocols to a machine-amenable format

The texts of biomedical protocols, like many types of text in natural language, may be ambiguous and contain errors. The automated processing of biomedical protocols has additional challenges:

• The protocol text could come in various file formats, such as txt, tex, doc, docx, pdf.

• The use of language differs between labs. That is usually due to lab or material specificity, and consequently some terms may have different implications in different labs. For example the term *overnight *may refer to 12 hours in some labs, 18 hours in others, and in some situations to be of no importance. The failure to capture such information accurately may result in the failure to obtain the desired experimental results. For example if an experiment requires the culturing a bacterial culture overnight, the experimental results may vary significantly depending on if the culture has been growing for <12 or >18 hours (bacteria can double in less than an hour).

• Biochemical entities may be referred to by different names. In order to disambiguate the biochemical names it is necessary to link each occurring in a protocol text biochemical entity to its unique ID from a commonly used external resource.

• Different experimental actions may have a varying number of descriptors some of which could be missing. This missing information, if essential for the execution of the protocol, must be captured and then processed.

Based on our extensive analysis of biomedical procedures, we have developed the following (semi-) automated framework for the translation of biomedical protocols expressed in natural language (English) into a machine-amenable semantically defined format:

1. *Input*: a biomedical protocol as text, EXACT2 as a reference model, and a list of semantic clues (e.g. '°C' appears with a value for the *descriptor: temperature*, 'in order to' appears with the value of the *descriptor: goal*).

2. *Input the laboratory-specific information*:

a. List of equipment (e.g. Thermo Scientific Forma Direct Heat CO2 Incubator TC 230, Incu-Shaker™ Mini).

b. List of biochemical entities (e.g. Herculase II Fusion DNA Polymerase, Dimethylsulfoxide).

c. Abbreviations (e.g. ON = 'overnight', RT = 'room temperature', DMSO = 'Dimethylsulfoxide').

d. List of default settings (e.g. ON = 16 hours, RT = 22°C).

3. *Process text*:

a. Convert text to a plain text format, e.g. txt.

b. Identify and normalize the named entities (NE) identified in the text.

c. Identify in the text nouns, verbs, and other parts of speech (POS).

d. Split the text into sentences.

4. *Identify experimental actions *in each sentence by matching the normalized verbs or verb phrases to the subclasses of the class EXACT2: *experimental action*. If a sentence contains several experimental actions, then create the corresponding number of copies of this sentence where each copy has only one experimental action.

5. *Identify descriptors *of each experimental action and the values of the descriptors by matching the descriptors defined in EXACT2 and using the semantic clues. If a descriptor defined in EXACT2 as essential has not been identified in the corresponding sentence, then request the user to input information about this descriptor and its value.

6. *Output *the list of identified experimental actions, their descriptors and the corresponding values.

7. *Verify *the output list of experimental actions and their descriptors with the user. The user should *correct errors *(if any) and/ or *confirm *that the translation is correct.

8. Manually *update *the input information if the user has made corrections or identified new experimental actions, e.g. a new synonym of an existing experimental action can be added to EXACT2.

This framework can be implemented in many ways. EXACT2 is encoded in a standard W3C language OWL-DL, but it can be easily translated into other formats, i.e. RDF, XML, java, or txt. Biochemical entities should be recorded along with their IDs to disambiguate these terms. Lab-specific internal IDs or IDs of the suppliers can be provided. However, it is recommended to use external IDs provided by commonly used resources like ChEBI whenever possible. There are also Biolexicons available to serve the purpose [21].It is harder to assign external IDs to equipment items. However there are projects that aim to semantically define laboratory equipment. For example the eagle-i project provides a national (US) research resource discovery platform that helps biomedical scientists search for laboratory resources [22].

There are various converters from various formats to the txt-format (see for example Zamzar converter [23]). There are many high-quality POS taggers. For example POS tagger CLAWS has consistently achieved 96-97% accuracy [24]. The National (UK) Centre for Text Mining (NaCTeM) provides various text mining tools, including GENIA Sentence Splitter (GeniaSS) optimized for biomedical texts [25]. We used Apache OpenNLP tools to process biomedical protocols we worked with (see [26]).

To illustrate the process of the identification of the experimental actions and their descriptors in the text, suppose we have the following sentences:

Adjust to 10% TCA.

Incubate at 30°C overnight.

**Figure 2:**
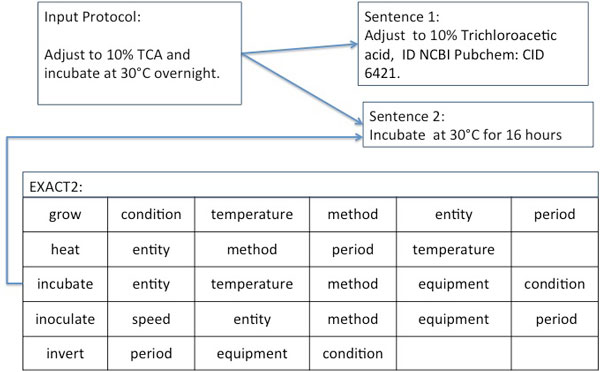
**The identification of experimental actions in the text**. The Translator engine searches the input text for experimental actions defined in EXACT2. The experimental actions *incubate *and *adjust *have been identified in this example protocol.

We assume that the text has been processed using appropriate text mining tools, and all NEs and POSs have been recognised and disambiguated. For example, TCA will be found in the text and checked against both the list of abbreviations and biochemical entities. The abbreviation will be replaced by the term *Trichloroacetic acid *and assigned with the ID NCBI Pubchem: CID 6421. All verbs then will be checked against subclasses of the class *EXACT2: experimental action*. The verb 'incubate' will be matched with the class *EXACT2 *000049: incubate and the verb 'adjust' will be matched with the class *EXACT2 000089*: *adjust *(see Figure 2). EXACT2 defines the following descriptors for the experimental action *incubate*:

• biochemical entity (linked to the experimental action *via *the relation *is-participant-of*),

• condition (linked to the experimental action *via *the relation *is-proposition*),

• temperature (linked to the experimental action *via *the relation *is-quality-of*),

• period (linked to the experimental action *via *the relation *is-proposition*), and

• optional descriptors: *equipment, protocol method, goal*.

**Figure 3:**
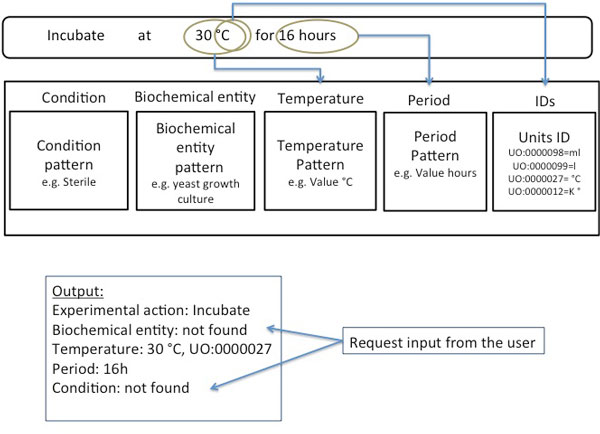
**The identification of descriptors of experimental actions in the text**. The Translator engine searches the text for the descriptors defined in EXACT2. Where possible values for the specified descriptors are extracted, and such ambiguous expressions as *overnight *are resolved. In this example the value of the descriptor *temperature *is 30°C and assigned with the ID: "UO:0000027" from the Units Ontology (see [39]).

A translating engine recognises the defined in EXACT2 descriptors in the given sentence. It is easy to recognize the value of the descriptor *temperature *by the clue '°C' and the value of the descriptor *period *as 16 hours (see Figure 3). However, the information about the participating biochemical entity and a condition is missing. This information is defined in EXACT2 as essential and therefore has to be specified. Therefore a translating engine will ask the user to input information about what is to be incubated, and under what condition. Thus all the essential information for the reproducibility of the protocol information will be captured and represented in a semantically defined form.

A more intelligent approach for the resolving of a missing descriptors issue would be to infer the missing values from previous actions, or to use default reasoning. The user should then be asked to check if the inference is correct. For example, we observed that a biochemical entity participating in an experimental action is often not specified in protocol text. However such an entity, e.g. 'yeast growth culture', can be easily identified from the preceding experimental actions. Also, information about typical scenarios, e.g. under what conditions an incubation with the use of the specified incubator for the specified biochemical entity typically takes place, can be captured and employed as default values.

The proposed framework ensures that biomedical protocols are translated to a machine-amenable format accurately, and EXACT2 and the supporting knowledge base of semantic clues are being continuously improved. Our evaluation suggests that at present EXACT2 supports the identification of 83-95% experimental actions in protocol texts (depending on if a protocol is from a research area that has been already used for the construction of EXACT2). The coverage of EXACT2 is improving through the analysis on new protocols. We propose that protocols translated to a machine amenable format should be deposited to public repositories for future re-use. Many operations on such protocols, like search, comparison and retrieval, will be enhanced and yield more accurate results.

## Future work: petri nets for the representation of experimental workflows

Ontologies are well suited for the representation of declarative and static domain knowledge, but they generally struggle with the representation of complex sequences of events unfolding in time. However there is a need in the integration of a rigorous logical representation of key experimental steps, with the temporal sequence of those steps, so as to capture the explicit semantics of whole experimental procedures.

There were several attempts to integrate EXACT with the representation of experimental workflows. In the original work on EXACT [7] we encoded protocols in Python. This approach to the representation of workflows was not popular with biologists. Maccagnan *et al *[27] developed the COW (Combining Ontologies with Workflows) software to formalize workflows built on ontologies. The method was specifically set up to support the design of structured protocols for biological laboratory experiments. The workflows were enhanced with ontological concepts taken from the developed domain-specific ontologies, including EXACT [25]. Unfortunately this project has now been abandoned (*personal communication*).

In order to adress the need for rigorous representation of both experimental actions and their sequence, we have recently experimented with using the Petri net formalism to representat workflows of experimental procedures, with encouraging results. Petri nets are used as a formal and graphically appealing language for modelling systems. They are suitable for the representation of biochemical reactions in metabolism, signal transduction and gene expression, and in reconstructing complex molecular networks. For example, Petri nets have been applied to the regulation of the *lac *operon [28]. Duchenne muscular dystrophy [29], the response of *S. cerevisiae *to mating hormones [30], and the yeast cell cycle [31].

A general Petri net has the following main components [32]:

• **Places **are passive nodes indicated by circles and refer to conditions or states. Places are allowed to carry tokens.

• **Transitions **are active nodes indicated by squares and describe state shifts, system events and activities. Transitions consume tokens from its pre-places and produce tokens within its post-places according to the arc weights.

• **Tokens **are variable elements indicated by dots within a place. Tokens may refer to a concentration level, a number of proteins, temperature, etc. Tokens are consumed and produced by transitions.

• Directed **arcs **are inactive elements visualised by arrows. Arcs specify the causal relationships between transitions and places and may have weights.

Petri net semantic describes the behaviour of nets [32]. We suggest to deepen the semantic representations of Petri nets by defining not only the behaviour of the system, but also the semantic meaning of each element of a Petri net representing an experimental workflow. Experimental actions can be encoded as transitions, the most essential descriptors of experimental actions such as time and vital experimental conditions as places may be marked with a token indicating whether or not the condition is fulfilled (true). Arcs can be used to represent the sequence of experimental actions.

**Figure 4:**
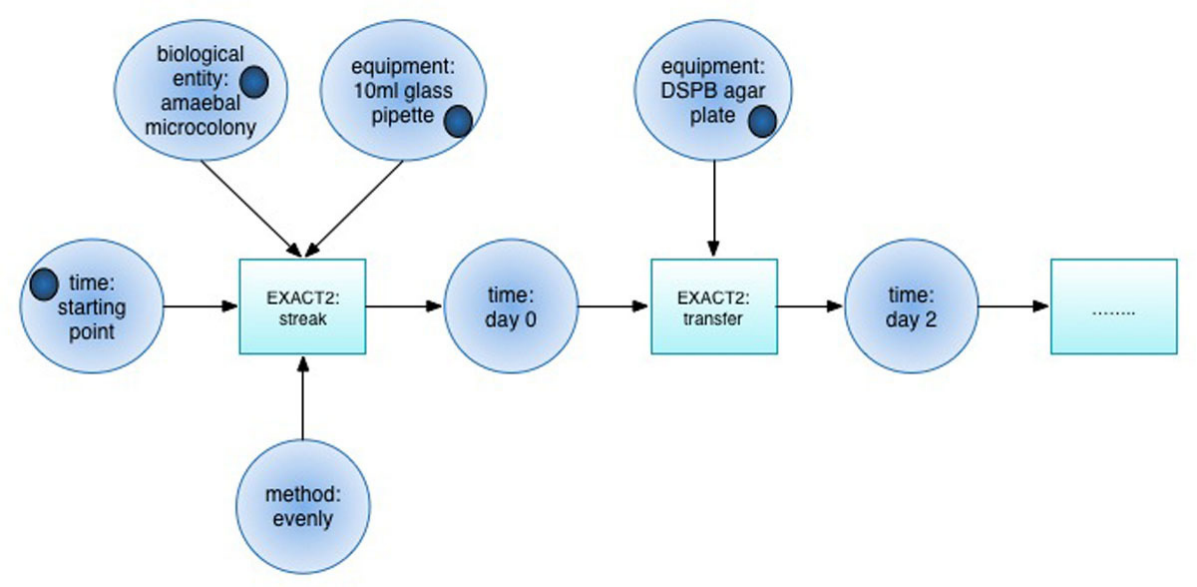
**An example of a Petri net (a fragment)**. The semantics of the places and transitions of this Petri net is defined through the use of EXACT2. Two experimental actions *streak *and *transfer*, defined in EXACT2, are used as labels to represent the semantics of the transitions. The descriptors of those experimental actions and their values, e.g. *equipment: 10 ml glass pipette *are used to represent the semantics of the places. The tokens (dark dots) indicate the necessary conditions for the transitions to take place.

For example, we considered the experimental procedures for isolation of *Physarum polycephalum *plasmodial mutants altered in sporulation obtained by chemical mutagenesis of flagellates [33].Corresponding genetic screens are run in the Magdeburg Centre for Systems Biology [34]. *Physarum polycephalum *belongs to the amoebozoa group of organisms. The experimental procedure is complex, non deterministic and takes ten days to complete (see the 'Growth of amoeba and preparation of flagellate suspension' procedure in the materials and methods section, [33]). Figure 4 shows a fragment of this procedure represented with a Petri net. The semantics of the places and transitions is defined with the use of the EXACT2 classes. The experimental actions, e.g. *streak, transfer*, are represented as transitions, and the key descriptors of those experimental actions are represented as places, e.g. *amoebae microcolony, DSPB agar plate *. In order to support the representation of such workflows, we will have to add to EXACT2 such descriptor as *time point*. Also, transition firing rules have to be defined appropriately in order to ensure the correct dynamic behaviour of the Petri net.

We suggest that an integration of the formalisms of Petri nets and EXACT2 will provide a powerful representation of experimental workflows. It would not only fully capture the semantics of experimental procedures, but also would enable one to simulate such procedures before physically executing them in the laboratory.

## Disscussion

A key insight of the scientific revolution was the importance of experimental results that could be reproduced in different laboratories [35]. What was true in the 17^th ^century is still true today in the 21^st ^century: repeatable experiments are a hallmark of science. It is widely accepted that for new knowledge to be published in a scientific journal the protocols used to derive that new knowledge must also be published. The protocols are necessary to reproduce the observations upon which the knowledge is based, and to enable this the protocols need to be completely specified.

Modern laboratory science requires the use of sophisticated protocols [36,37]. However, these are still generally expressed using natural language, and unfortunately use of natural language inevitably introduces ambiguities about how to reproduce experiments. The result is the failure to reproduce results, with the subsequent loss of time and money. While working on this paper we noticed that yet another research paper has been retracted from *Nature *because results could not be reproduced. Obokata *et al*. [38] in their paper titled 'Stimulus-triggered fate conversion of somatic cells into pluripotency' reported on a cheap and quick method of producing stem cells. The Reuters news agency reports Prof. Wakayama of the University of Yamanashi told Japanese TV: "When conducting the experiment, I believed it was absolutely right... But now that many mistakes have emerged, I think it is best to withdraw the research paper". We argue that if experimental procedures reported by [38] were expressed with the use of EXACT2 then mistakes could be identified earlier and other groups could reproduce their results more easily.

## Conclusions

In this paper we present a fundamentally new version of the ontology EXACT2 designed to support the accurate and computer friendly recording of information about biomedical procedures. EXACT2 follows best practice in ontology development, and the recommendations of the OBO Foundry. It can therefore be directly integrated with other bio-medical ontologies.

We present a framework for the translation of biomedical protocols from natural text to a machine amenable semantically-defined format. The proposed framework employes EXACT2 as a reference model to identify experimental actions and their descriptors in protocol texts, and assigns them unique IDs.

We also demonstrate that the integration of EXACT2 with the formalism of Petri nets will enable the capture of explicit semantics of experimental workflows, and validate the workflow through simulations of the experimental procedure. We suggest that biomedical protocols represented in a formal machine friendly way should be submitted to public repositories for future re-use.

## List of abreviations used

EXACT - an ontology of EXperimental ACTions; GLP - Good Laboratory Practice; GMP - Good Manufacturing Practice; OBI - the Ontology for Biomedical Investigations; BSA - Bovine Serum Albumin; OBOF - Open Biomedical Ontologies Foundry; MIREOT - Minimum Information to Reference External Ontology Terms; BFO - the Basic Formal Ontology; IAO - the Information Artifact Ontology; PATO - Phenotype And Trait Ontology; ChEBI - Chemical Entities of Biological Interest; SUMO - the Suggested Upper Merged Ontology; OED - Oxford English Dictionary; URI - Unique Resource Identifier; ID - Identifier; ON - Overnight; RT - Room Temperature; DMSO - DiMethylSulfOxide; NE - Named Entities; POS - Part Of Speech.

## Competing interests

The authors declare that they have no competing interests.

## Authors' contributions

LNS is the leader of the EXACT2 project. She contributed to the ontology development and the use case. DN initiated the work on the translation of biomedical protocols from natural text to machine amenable form. He contributed to the analysis of procedures. RDK worked on the ontology development and manuscript. PSB worked on textual definitions and descriptors of experimental actions defined in EXACT2. EH analysed protocols and worked on the use case. VB and NJS contributed to the analysis of protocols. WM contributed to the formal representation of experimental procedures and workflows. BBR contributed to the development of EXACT2 and the use case.

## Declaration

Publication costs for this article were funded by the corresponding author's institution.

This article has been published as part of *BMC Bioinformatics *Volume 15 Supplement 14, 2014: Semantic, social, mobile applications for bioinformatics and biomedical laboratories: selected works from the 13th International Workshop on Network Tools and Applications in Biology (NETTAB 2013). The full contents of the supplement are available online at http://www.biomedcentral.com/bmcbioinformatics/supplements/15/S14.
